# Bariatric surgery: to bleed or not to bleed? This is the question

**DOI:** 10.1186/s12893-022-01783-w

**Published:** 2022-09-04

**Authors:** Giovanna Pavone, Alberto Gerundo, Mario Pacilli, Alberto Fersini, Antonio Ambrosi, Nicola Tartaglia

**Affiliations:** Department of Medical and Surgical Sciences, University of Foggia, Viale Pinto 1, 71122 Foggia, Italy

**Keywords:** Bariatric surgery, Bleeding, Hemorrhage, Sleeve gastrectomy, Roux-n-Y gastric bypass

## Abstract

**Background:**

Bariatric surgery procedures are the most successful and durable treatment for morbid obesity. Hemorrhage represents a life-threatening complication, occurring in 1.3–1.7% of bariatric surgeries.

**Materials and methods:**

We examined patients undergoing Bariatric Surgery from July 2017 to June 2020 (Group A) and those operated from July 2020 to June 2022 (Group B) in our Department. Starting from July 2020 we have implemented intraoperative measures to prevent postoperative bleeding, increasing mean arterial pressure (MAP) by 30% compared to preoperative and reducing the pneumoperitoneal pressure of CO_2_ to 8 mmHg in the last 15 min of the operation.

**Results:**

The study gathered 200 patients divided into the two described groups. The mean age of Group A is 44 ± 8.49 and 43.73 ± 9.28. The mean preoperative BMI is 45.6 kg/m^2^ ± 6.71 for Group A and 48.9 ± 7.15 kg/m^2^ for Group B. Group A recorded a mean MAP of 83.06 ± 18.58 mmHg and group B a value of 111.88 ± 12.46 mmHg (p value < 0.05 and *z*-score is 4.15226 and the value of U is 13,900). We observed 9 cases of bleeding in group A, most of them being treated with medical therapy and transfusions; only 1 hemodynamically unstable patient underwent re-laparoscopy. We reported only 2 cases of bleeding in group B, one of which required blood transfusions.

**Conclusion:**

From our study we can conclude that increasing mean arterial pressure (MAP) by 30% compared to preoperative and reducing the pneumoperitoneum pressure of CO_2_ to 8 mmHg in the last 15 min of the operation led to a decrease in bleeding cases in group B and, most importantly, all the bleedings were easily controllable with medical therapy and/or transfusions. These measures allowed us to reduce postoperative bleeding.

## Introduction

Obesity is classified as one of the most severe global public health problems. Over 2.1 billion adults worldwide are considered overweight or obese; 640 millions of these are classified as obese. Bariatric surgery has proven to be an effective treatment strategy in treating obesity and improving associated comorbidities. At present, the most commonly conducted bariatric surgical procedures are Roux-en-Y gastric bypass (RYGB) and Sleeve gastrectomy (SG) [1].

Consensual indications for bariatric surgery are BMI ≥ 40 kg/m^2^, BMI ≥ 35 kg/m^2^ with T2D, or other comorbidities that could be significantly improved after bariatric surgery.

Bleeding, leakage, and gastric fistulae are the most common intraoperative complications and post-operative complications after bariatric procedures [2].

Literature reports a wide range of post-bariatric surgery complications, from 1 to 29%. Most common postoperative complications reported include leakage, hemorrhage, fistula, surgical site infection, abscess, gastric dilatation, stricture, wound complication, and nutritional deficiencies [3–5]. Hemorrhage represents a life-threatening complication, occurring in 1.3–1.7% of bariatric surgeries.

Jan Mulier and Bruno Dillemans studied the effect of systolic blood pressure on gastric suture hemorrhage during laparoscopic gastric bypass surgery [6–8], we created and introduced in 2020 a Protocol for the Prevention of Postoperative Bleeding after Laparoscopy Gastrectomy sleeve. According to this protocol, mean arterial pressure (MAP) is raised intraoperatively by 30% compared to the preoperative level and the reduction of the CO_2_ pneumoperitoneum pressure to 8 mmHg in the last 15 min of the operation [9–11].

The aim of this study is to evaluate whether increasing mean arterial pressure (MAP) by 30% compared to preoperative and reducing the pneumoperitoneal pressure of CO2 to 8 mmHg in the last 15 min of the operation could reduce postoperative bleeding after laparoscopic sleeve gastrectomy (LSG).

## Materials and methods

This is a retrospective study that includes patients undergoing Laparoscopic Sleeve Gastrectomy all performed by the same experienced surgeon from July 2017 to June 2020 (Group A) and those operated from July 2020 to June 2022 (Group B) in our Department [12, 13]. Starting from July 2020 we have implemented intraoperative measures to prevent postoperative bleeding, increasing mean arterial pressure (MAP) by 30% compared to preoperative (98 ± 10 mmHg) and reducing the pneumoperitoneal pressure of CO_2_ to 8 mmHg in the last 15 min of the operation. Preoperative patients’ characteristics, including sex, age, BMI and preoperative comorbidities, type 2 diabetes (T2D), hypertension (HTN), obstructive sleep apnea (OSA). We consider also length of stay, operative time, rate of conversion to open surgery. We used the following symptoms to diagnose early postoperative bleeding: orthostatic hypotension, dizziness, hematemesis, melena, hypotension (< 90/60 mmHg), tachycardia (> 120 bpm). Patients who were hemodynamically unstable or did not respond well to the packed red blood cell (PRBC) transfusion underwent reoperation.

### Eligibility criteria

Adult patients of both sexes aged 18–65 years with morbid obesity defined as BMI > 40 kg/m^2^ or BMI > 35 kg/m^2^ with at least one associated major comorbidity were included, underwent laparoscopic Sleeve Gastrectomy. We excluded patients with secondary obesity due to endocrine and psychological disorders, patients under antiaggregant and anticoagulant therapy and re-do surgery.

### Statistical analysis

Continuous data were expressed as mean and standard deviation (SD) and they were analyzed with Chi-square test with a p value less than 0.05 (p < 0.05) for statistical significance and Mann–Whitney *U* test.

### Surgical technique

The technique involves the use of 4 12 mm trocars. Pneumoperitoneum is induced by a 0° optical trocar and maintained at 15 mmHg. The first trocar is usually inserted along the left mid-clavicular line approximately 3 fingers from the costal arch, another trocar along the left axillary line, a third trocar 1 cm to the right of the midline, and the fourth trocar along the right mid-clavicular line. A 10 mm, 30° laparoscope is used.

The left lobe of the liver is retracted to expose the lesser gastric curvature and the gastroesophageal junction. The procedure begins by dissecting the small branches of the gastroepiploic arch 6 cm from the pylorus. The dissection continues along the great curvature of the stomach, remaining very close to the gastric wall, up to the short gastric vessels which are also dissected. The stomach is then raised to expose its posterior wall and the adhesions are lysed. His angle is fully mobilized and the left diaphragmatic pillar exposed. The gastric tubule is created on the guide of a 40 F Bugie using mechanical suturing machines with charges of different thickness depending on the thickness of the gastric wall. At this point the bougie is removed and the resected stomach is extracted from the abdomen through the mesogastric access.

The pneumoperitoneal pressure of CO_2_ is reduced to 8 mmHg, the haemostasis is checked, in case of bleeding we proceed with cauterization with monopolar forceps or with the use of laparoscopic hemostatic agents. Abdominal drainage is placed.

## Results

The study gathered 200 patients divided into the two described groups. The mean age of Group A is 44 ± 8.49 and 43.73 ± 9.28 for Group B. The mean preoperative BMI is 45.6 ± 6.71 kg/m^2^ for Group A and 48.9 ± 7.15 kg/m^2^ for Group B. Group A included 23 patients with Type 2 diabetes (T2D), 61 with Hypertension (HTN) and 11 with Obstructive sleep apnea (OSA); while in group B 15 suffered from T2D, 52 from HTN and 9 from OSA. Mean length of stay (LOS) was 3.65 ± 1.83 days for Group A and 3.27 ± 1.01 days for Group B; mean operative time (OT) was 59.96 ± 8.62 min for Group A and 59.57 ± 5.96 min for Group B. There were no conversions to open surgery in both groups. Group A recorded a mean MAP of 83.06 ± 18.58 mmHg and group B a value of 111.88 ± 12.46 mmHg (p value < 0.05 and *z*-score is 4.15226 and the value of U is 13,900). We observed 9 cases of bleeding in group A and only 2 cases of bleeding in group B (Tables 1, 2).

**Table 1:** Demographic and operative characteristics of the study groups

	Group A	Group B
Age (years) mean ± (SD)	44 ± 8.49	43.73 ± 9.28
Preoperative BMI (kg/m^2^) mean ± (SD)	45.6 ± 6.71	48.9 ± 7.15
Mean Arterial Pressure (mmHg) mean ± (SD)	83.06 ± 18.58	111.88 ± 12.46

*SD* standard deviation

**Table 2:** Operative characteristics of the study groups

	Group A	Group B	p-value	*z*-score
Length of stay (days) ± (SD)	3.65 ± 1.83	3.27 ± 1.01	0.36282	0.91138
Operative Time (minutes) ± (SD)	59.96 ± 8.62	59.57 ± 5.96	0.3843	0.87351
Rate of conversions to open surgery number (%)	0 (0%)	0 (0%)	n.s	n.s
Intraoperative Mean Arterial Pressure (mmHg) mean ± (SD)	83.06 ± 18.58	111.88 ± 12.46	0.011847	4.15226
Bleeding number (%)	9 (9%)	2 (2%)	n.s	n.s

*SD* standard deviation

## Discussion

According to the literature, 3.1–8.8% of patients have early postoperative complications [14–17] and 0.9–9.4% of patients return to the operating room [18, 19]. Postoperative hemorrhage is one of the most common early complications occurring both intraluminal and intra-abdominal (i.e., from the staple line, omentum, port sites, or damage to the liver or spleen).

Early postoperative bleeding is linked to longer hospitalization, major complications (such as sepsis and organ failure), reoperation and mortality [20–23]. Therefore, there is a need for preventive strategies in bariatric surgery due to the severe consequences of early postoperative.

In our study we have implemented intraoperative measures to prevent postoperative bleeding, increasing mean arterial pressure (MAP) by 30% compared to preoperative and reducing the pneumoperitoneal pressure of CO2 to 8 mmHg in the last 15 min of the operation; the difference between Intraoperative MAP of two group is statistically significant. While, in our study, LOS and OT do not affect the incidence of bleeding as the differences between the two groups are not statistically significant.

In the patients of 2 group with HTN, high blood pressure was completely managed before surgery, and they took their antihypertensive medications in the morning of surgery. Prior searches found a correlation between HTN and early postoperative bleeding [18, 24, 25]. But the lack of association in the current study may be related to the well- controlled blood pressure before surgery.

We observed 9 cases of bleeding in group A, treated with medical therapy and packed red blood cell (PRBC); only 1 hemodynamically unstable patient underwent re-laparoscopy and placement of clips at the source of bleeding. We reported only 2 cases of bleeding in group B, only one of which required PRBC.

We have noticed that patients who suffered post operative bleeding were featured by lower blood pressure values in comparison with those belonging to the control group during the critical window toward the end of LSG.

We have come to this conclusion considering that bleeding patients were relatively hypotensive at the time of the final abdominal inspection and consequently we were not able to recognize dormant areas which may have bled if blood pressure parameters were closer to their normal ranges [26–29].

In the literature there are very few studies that analyze post-operative bleeding after LSG, only 2 studies consider the increasing of the mean arterial pressure (MAP) by 30% and none the reducing the pneumoperitoneal pressure of CO2; Banescu et al. has shown that the intraoperative risen of the blood pressure (BP) with 30% helps identifying and controlling the bleeding sources thus reducing the incidence of postoperative bleeding in LSG [30]. Ying et al. concluded in their study that postoperative haemorrhage requiring transfusion in bariatric surgery patients who, at the time of closure, do not appear to have any evidence of bleeding is a rare but challenging complication that patients with postoperative bleeding were relatively assumptions regarding their baseline blood pressure during a critical window of surgery when the operation is complete and the abdomen is inspected for potential bleeding from the operating table [22].

The result of the persistance of the pneumoperitoneum is an increase in intra-abdominal pressure (IAP).

The compliance of the abdominal wall (Cab) is described as the ease of the abdominal expansion and it’s determined by the parietal and diaphragmatic elasticity. The measure of abdominal compliance is the change in intra abdominal volume (IAV) per change in IAP.

Abdominal compliance determines the limits of IAP and the volume of gas necessary to reach the maximal intraperitoneal space for laparoscopy [31, 32].

The induction of a intra abdominal pressure exceeding the limits of Cab determines a reduction in blood flow, perfusion and urinary output, leading to hypoxia, ischemia and increasing oxidative stress.

From these informations it can be deduced that by reducing the pneumoperitoneal pressure of CO2 during surgery, hidden bleeding become evident.

## Conclusion

From our study we can conclude that increasing mean arterial pressure (MAP) by 30% compared to preoperative and reducing the pneumoperitoneum pressure of CO_2_ to 8 mmHg in the last 15 min of the operation led to a decrease in bleeding cases in group B and, most importantly, all the bleedings were easily controllable with medical therapy and/or transfusions. These measures allowed us to reduce postoperative bleeding. This protocol can be a great place to start but future prospective studies will be required to determine if normalizing blood pressures intraoperatively and reducing pneumoperitoneum pressure of CO_2_ is safe and can decrease the incidence of postoperative hemorrhage.

## Data Availability

All data generated or analysed during this study are included in this published article.
